# Plasma and Urine Pharmacokinetics of Oral Fosfomycin Tromethamine in Dogs

**DOI:** 10.3390/vetsci10060391

**Published:** 2023-06-08

**Authors:** Nattha Jariyapamornkoon, Koranis Patthanachai, Nipattra Suanpairintr

**Affiliations:** 1 Graduate Program in Veterinary Bioscience, Faculty of Veterinary Science, Chulalongkorn University, Bangkok 10330, Thailand; nattha.j@student.chula.ac.th; 2 Department of Pharmacology, Faculty of Veterinary Science, Chulalongkorn University, Bangkok 10330, Thailand; koranit.p@chula.ac.th

**Keywords:** dogs, Fosfomycin tromethamine, liquid chromatography tandem mass spectrometry (LC- MS/MS), plasma, pharmacokinetic, urine

## Abstract

**Simple Summary:**

Fosfomycin tromethamine, an oral formulation of Fosfomycin, is the most commonly used formulation in humans due to its improved oral bioavailability. However, the information on Fosfomycin tromethamine in dogs is limited. Thus, this study aimed to determine the pharmacokinetic parameters of oral Fosfomycin tromethamine in canine plasma and urine using liquid chromatography tandem mass spectrometry (LC-MS/MS). Six healthy male beagle dogs underwent a three-period three-treatment study: treatment 1 and 2 with single oral Fosfomycin tromethamine at 40 and 80 mg/kg (the total doses with tromethamine salt were 75 and 150 mg/kg, respectively) and treatment 3 with intravenously Fosfomycin disodium at 57 mg/kg (the total dose with disodium salt was 75 mg/kg). The results indicated that, in dogs, oral Fosfomycin as the tromethamine salt was better absorbed into the blood circulation compared to disodium salt as previously reported. The Fosfomycin concentrations in urine were much higher than those in plasma (>100 fold). There were no serious adverse effects except loose stool in some dogs. These results suggest that Fosfomycin tromethamine could serve as a viable substitute for oral antibiotics in bacterial cystitis treatment in dogs with multidrug resistant infections when other antibiotics have failed.

**Abstract:**

Fosfomycin is a broad-spectrum, bactericidal antibiotic with low toxicity. It has been used in human medicine and is a promising candidate for treating infections in veterinary medicine. Different Fosfomycin salts exhibit various degrees of bioavailability. Tromethamine salt is the most commonly used oral form due to its improved bioavailability. However, information regarding its use with dogs is limited. Therefore, this study aimed to investigate the pharmacokinetics of oral Fosfomycin tromethamine in canine plasma and urine using liquid chromatography tandem mass spectrometry (LC-MS/MS). Six healthy male beagles underwent a three-period three-treatment study: treatment 1 and 2 with single oral Fosfomycin tromethamine at 40 and 80 mg/kg (the total doses with tromethamine salt were 75 and 150 mg/kg, respectively), and treatment 3 with intravenously Fosfomycin disodium at 57 mg/kg (the total dose with disodium salt was 75 mg/kg). Dogs receiving oral Fosfomycin tromethamine at 75 and 150 mg/kg, maximal drug concentration (Cmax) in plasma produced results of 34.46 ± 12.52 and 66.40 ± 12.64 µg/mL, oral bioavailability (F) was approximately 38 and 45%, while urine Cmax was 4463.07 ± 2208.88 and 8784.93 ± 2303.46 µg/mL, respectively. No serious adverse effects were reported, except loose stool in some dogs. The tremendously high urine Fosfomycin concentrations indicate that oral Fosfomycin tromethamine is suitable as an alternative treatment for bacterial cystitis in dogs.

## 1. Introduction

Fosfomycin is a phosphonic acid derivative antibacterial drug [1]. This drug has been categorized in its own class due to its unique chemical structure [2]. Fosfomycin inhibits the early process of bacterial cell wall synthesis by inactivation of UDP-N-acetylglucosamine-3-enolpyruvyl transferase (MurA) [3]. MurA is a necessary enzyme in the production process of UDP-N-acetylmuramic acid (UDP-MurNAc), the peptidoglycan precursor [2,3,4]. Fosfomycin is a bactericidal drug with broad spectrum activities against both gram positive and negative bacteria [5,6,7,8], including ESBL-producing and multidrug resistant (MDR) bacteria [8,9,10]. Fosfomycin resistant bacteria tend to have a slower growth rate and a weaker virulence when compared with its wildtype strains [11].

Fosfomycin has a small molecular weight (138.06 g/mol) and good water solubility [12,13]. At present, three different formulations of Fosfomycin are available worldwide: (1) Fosfomycin tromethamine (granules for oral solution), (2) Fosfomycin calcium (oral capsules) and (3) Fosfomycin disodium (IV solution). With regard to oral administration, Fosfomycin is hydrolyzed in acidic environment of the stomach, resulting in low absorption and bioavailability [14,15]. To counteract this issue, the use of tromethamine salt, an alkalizer, has been proposed, in order to increase the pH levels and alkalinity which may help to delay acid-catalyzed hydrolysis from gastric acid and improve bioavailability. Thus, Fosfomycin tromethamine has been suggested as an oral formulation [16]. In humans, Fosfomycin tromethamine (trometamol) is rapidly absorbed with absolute bioavailability of approximately 33–53% [17,18,19]. This drug has negligible plasma-protein binding (<0.5%) [20,21] and is widely distributed in tissue and fluid [22]. Without metabolic transformation, 40–50% of Fosfomycin is excreted as unchanged drug via urine [17,18,23,24], and Fosfomycin tromethamine is recommended for uncomplicated urinary tract infection (UTI) treatment in humans [24,25]. Fosfomycin is a safe drug with low toxicity [1,26]. Adverse effects such as loss of appetite and diarrhea have been reported in dogs [11,27,28], whereas diarrhea is the most common adverse effect in humans [24,25,29]. Based on its properties, Fosfomycin may be considered as an interesting alternative drug for treating canine bacterial cystitis, especially for infections with no other antibacterial choices.

Fosfomycin is an old antibacterial drug, which is included in the World Health Organization (WHO) list as a critically important antimicrobial (CIA) [30]. The Antimicrobial Advice Ad Hoc Expert Group (AMEG) of the European Medicines Agency (EMA) classified Fosfomycin in Category A (avoid) due to concerns for public health related to increased antimicrobial resistance. Based on Category A (avoid), Fosfomycin is not currently authorized as a veterinary medicine in the EU and may only be administered to individual companion animals under exceptional circumstances [31,32]. These conditions may be some of the reasons why Fosfomycin is not frequently used in clinics. The information about Fosfomycin in animals is very scarce. However, this drug has been recommended to be reserved for canine bacterial cystitis with multidrug resistance infection when no alternative drugs are available [27,33]. Pharmacokinetic studies in dogs should be conducted to determine the effective dose for treating the infection.

From their early study in dogs, the authors investigated the plasma PK of Fosfomycin sodium at 40 and 80 mg/kg [20] and the urine PK of Fosfomycin at 80 mg/kg [34]. However, a PK study of oral Fosfomycin tromethamine, a recommended oral formulation for bacterial cystitis, in both plasma and urine samples, has not been reported. Therefore, the objective of this study was to determine the plasma and urine concentrations, as well as the pharmacokinetic parameters, of a single oral Fosfomycin tromethamine at dosages of 40 and 80 mg/kg (the total dose with tromethamine salt was 75 mg/kg, respectively) in dogs. The appropriate liquid chromatography tandem mass spectrometry (LC-MS/MS) protocols for both sample types were developed and validated.

## 2. Materials and Methods

### 2.1. Animal Procedure

Six healthy male beagle dogs (5.7 ± 2.5 years) with an average body weight (BW) of 12.7 kg (ranging from 10.3–15.1 kg) were used in this study. All dogs were healthy based on physical examination, complete blood count, serum analysis and urinalysis 48 h before the study and had no history of antimicrobial treatment for at least one month prior to the study. The subjects were housed in individual cages. In order to alleviate the discomfort associated with the collection of urine samples, a local anesthetic drug (topical 2% lidocaine gel) was applied during the urinary catheterization procedure. Furthermore, during this procedure, all dogs were carefully monitored for any signs of distress. If any problems were observed, the dogs would receive appropriate treatment. The animal experiment was conducted at the Veterinary Student Training Center, Nakorn Pathom, Faculty of Veterinary Science, Chulalongkorn University. The procedures were reviewed and approved by IACUC, the Ethics Committee of the Faculty of Veterinary Science, Chulalongkorn University (protocol number: 1931034).

All dogs were assigned to a three-period three-treatment study with six dogs per treatment; (1) Fosfomycin tromethamine 40 mg/kg (the total dose with tromethamine salt was 75 mg/kg) per oral (PO), (2) Fosfomycin tromethamine 80 mg/kg (the total dose with tromethamine salt was 150 mg/kg) PO, and (3) Fosfomycin disodium 57 mg/kg (the total dose with disodium salt was 75 mg/kg) IV (reference for bioavailability) with a 7-day washout period between each treatment, based on a Fosfomycin elimination half-life of 2.5 ± 1.1 h [11]. Six dogs in each treatment group received the same drug administration and had a minimum of 7 days of rest before the next treatment. To mitigate the risk of cumulative toxicity from Fosfomycin, kidney and liver function of all dogs in the three treatment groups were assessed through serum analysis and urinalysis before and after drug administration.

Fosfomycin tromethamine (Monurol^®^, Zambon, Cadempino, Switzerland), a granule drug, was dissolved in drinking water before immediately being fed to each dog for at least 30 min before a meal, while Fosfomycin disodium (Fosmicin^®^, Meiji, Tokyo, Japan) was intravenously injected through a cephalic venous catheter. All dogs were observed for adverse effects at least 2 days after each treatment and evaluated for complete blood count, serum analysis and urinalysis before and after each treatment.

Three ml of whole blood was collected through the cephalic vein at 0, 15, 30, 45 min and 1, 2, 3, 4, 6, 8, 12, 24, 36 and 48 h after Fosfomycin administration. Blood samples from each time point were transferred into EDTA-coated tubes and kept in a cold container at 4 °C before being centrifuged at 1500× *g* for 15 min at 4 °C. Plasma samples were harvested and transferred into sterile cryovials and stored at −80 °C for further analysis.

For urine samples, 10 mL were collected using sterilized urinary catheters with lidocaine 2% gel at 0, 30 min and 1, 2, 3, 4, 6, 8, 12, 24, 36 and 48 h after drug administration. Following urine collection at each time point, the urinary bladder was emptied to ensure fresh urine for the next time point. Urine samples were placed in cold containers at 4 °C before being vortexed, and the supernatants were aliquoted into sterile cryovials and stored at −80 °C.

Both plasma and urine samples were subjected to Fosfomycin concentration determination using LC-MS/MS and analyzed within two weeks. Fosfomycin concentrations of plasma and urine samples were conducted by Chula Pharmacokinetic Research Center, Faculty of Medicine, Chulalongkorn University.

### 2.2. LC-MS/MS Analysis

#### 2.2.1. Chemicals and Reagents

Fosfomycin trometamol CRS and tadalafil were reference standard based on European pharmacopoeia and USP, respectively. Other reagents and chemicals, acetonitrile (HPLC grade), methanol (HPLC grade) and ammonium hydroxide (AR grade), were purchased from Merck & Co., Rahway, New Jersey, NJ, USA., except for ammonium formate (AR grade), purchased from Sigma-Aldrich, Taufkirchen, Germany.

#### 2.2.2. Preparation of Solutions

Stock standard solution of Fosfomycin was produced by dissolving an appropriate amount of Fosfomycin in ultrapure water to a final concentration of 10,000 µg/mL for the calibration curve and quality control (QC) samples. The stock standard solution was further diluted with methanol to prepare eight working standard solutions for the calibration curve of plasma and urine.

For stock standard solution of tadalafil (as internal standard, IS), an appropriate amount was dissolved in methanol to a concentration of 1000 µg/mL. The stock standard solution was then diluted with methanol to prepare working standard solutions at 50 µg/mL.

Both of the stock standard solutions were stored in a refrigerator at 0–8 °C and protected from light.

Ten millimolar of ammonium formate solution was prepared by weighing 0.63 g of ammonium formate and dissolving in 1000 mL of ultrapure water. The solution was adjusted to a pH of 7.5 with ammonium hydroxide.

#### 2.2.3. Instruments and Chromatographic Conditions

Chromatographic separation was operated using a high-performance liquid chromatography (HPLC) system equipped with a mass spectrometer (LCMS 8040: Shimadzu Corporation, Kyoto, Japan). The compounds were separated on a Luna C18 column (150 × 4.6 mm, 5 µm, Phenomenex, Torrance, California, CA, USA) with an Inertsil ODS-3 C18 guard column (4.0 × 10 mm, 5 µm, GL Science, Tokyo, Japan) and maintained at 40 °C. The mobile phase system consisted of the mixture of acetonitrile/methanol (50:50, *v*/*v*) and 10 mM ammonium formate (pH 7.5) in a 60:40 ratio (*v*/*v*). The extracted samples were maintained in an auto-sampler at 15 °C and injected through a LC-MS/MS system at 5 µL and 1 µL for plasma and urine, respectively.

The analytes were detected with a triple quadrupole mass spectrometer equipped with an electrospray ionization (ESI) source spectrometer that was operated in the multiple reaction monitoring (MRM) negative mode. The MRM transitions of Fosfomycin were *m*/*z* 137.15 > 62.90 for plasma and 137.15 > 78.95 for urine and IS was *m*/*z* 390.10 > 135.00. The software program, Lab solution version 5.82 SP1 and Class agent software version 2.33SU3 were utilized for data analysis.

#### 2.2.4. Calibration Curve and Quality Control Samples

For plasma samples, the calibration curve was freshly prepared in each batch run. Eight series of calibration curve were prepared by spiking 10 µL of each working standard solution in 90 µL of blank plasma to reach the concentrations of 0.05, 1.0, 5.0, 10.0, 20.0, 40.0, 80.0 and 140.0 µg/mL, and 15 µL of IS. In urine, eight series of calibration curves were prepared by spiking 5 µL of each working standard solution in 45 µL of blank plasma to reach the concentrations of 1.0, 2.5, 5.0, 25.0, 100.0, 250.0, 500.0 and 1000.0 µg/mL, and 3 µL of IS. The concentrations of the samples were calculated from the linear equation by using regression analysis of the calibration curve as a weighting factor (1/C2).

The QC samples of plasma were prepared to reach concentrations at 0.05 µg/mL for the lower limit of quantification (LLOQ), 0.75 µg/mL for low quality control (LQC), 16.5 µg/mL for lowest medium quality control (Lowest MQC), 55.0 µg/mL for medium quality control (MQC) and 110.0 µg/mL for high quality control (HQC). The QC samples of urine were prepared to reach concentrations at 1.0 µg/mL for lower limit of quantification (LLOQ), 3.0 µg/mL (LQC), 350.0 µg/mL (MQC) and 750.0 µg/mL (HQC).

#### 2.2.5. Sample Extraction

The samples were extracted using protein precipitation by adding the mixture of acetonitrile/methanol (80/20, *v*/*v*) into 100 µL of plasma samples and 50 µL of urine samples to make a total volume of 250 µL and 300 µL, respectively, then mixed with the vortex mixer for 1 min. The mixtures were then centrifuged at 10,000 rpm, 10 °C for 5 min, then filtrated for the supernatant through a nylon syringe filter, pore size 0.22 µm, into a vial and placed in auto-sampler tray for the LC-MS/MS procedures.

#### 2.2.6. Method Validation

Method validation procedures were modified and performed by following the Guidance for Industry, Bioanalytical Method Validation (U.S. Department of Health and Human Services, FDA) [35] and the Guideline on Bioanalytical Method Validation (European Medicines Agency, EMA) [36]. The method validations consisted of selectivity, linearity, accuracy and precision, recovery, dilution integrity, carry-over, freeze and thaw stability, and post-preparative stability.

Selectivity was assessed by screening six different sources of blank plasma and urine to evaluate the interfering components at the retention time for Fosfomycin and IS.

For linearity, eight standard points were used to display calibration curves. The calibration curves ranged from 0.05–140.0 µg/mL for Fosfomycin in plasma and from 1.0–1000 µg/mL for Fosfomycin in urine. The coefficient of determination (R^2^) should be more than 0.99. The reproducibility for the calibration curve was evaluated on different days (*n* = 3).

Accuracy and precision were determined for LLOQ, LQC, MQC, and HQC samples for five replicates in each concentration. The inter batch for accuracy and precision was conducted on 3 different days.

The recovery was assessed by comparing the response of Fosfomycin for LQC and HQC samples from five replicates of pre-extracted samples with the response obtained from the post-extraction.

The dilution integrity was conducted to demonstrate that a sample with very high concentration (above ULOQ) can be diluted to a concentration within the working range and still provide a reliable result. The method was tested by preparing five replicates of twice the concentration of HQC, then diluted with a blank of the sample at 1:5 for plasma samples and 1:10, 1:20 and 1:50 for urine samples. The samples were analyzed and calculated by using the dilution factor.

Carry-over was examined by injecting the extracted blank samples after the extracted ULOQ.

The freeze and thaw stability were performed by preparing the samples at LQC and HQC, then three aliquots of each QC were analyzed as the freshly prepared samples. The QC samples were stored at −70 °C and thawed at room temperature. The freeze–thaw cycle was repeated three times for plasma samples and five times for urine samples and reanalyzed to compare the concentrations with the freshly prepared samples.

The post-preparative stability was conducted by preparing three replicates of LQC and HQC samples and determined with freshly prepared samples, then kept in the auto-sampler at 15 °C. After 24 h in the auto-sampler, the samples were evaluated again and compared with the freshly prepared samples.

### 2.3. Pharmacokinetic Analysis

The plasma PK parameters were analyzed using one-compartment model with PKanalix (PKanalix Suite 2021R2, Lixoft, Antony, France). The best fit model for IV route was the IV bolus administration, with no delay, one compartment distribution, and linear elimination. Similarly, for the oral route, the best fit model was oral/extravascular administration, with no delay, one compartment distribution, and linear elimination.

The percentages of bioavailability (*F*) of oral Fosfomycin (40 and 80 mg/kg *PO*) to the reference dose (57 mg/kg IV Fosfomycin disodium) were calculated based on the *AUC* 0–∞ (the linearity from the same Cl for *PO* and IV route) with the *following equation*:

$$F=\frac{\left(AUC\;PO)\times (Dose\;reference\right)}{\left(AUC\;reference)\times (Dose\;PO\right)}\times 100$$

where *AUC PO* was the *AUC* of the dose administered per oral, *AUC reference* was the *AUC* of the reference dose or dose administered IV, *Dose reference* was the dose administered IV (Fosfomycin disodium at 57 mg/kg), and *Dose PO* was the dose administered orally (Fosfomycin tromethamine at 40 and 80 mg/kg, respectively).

The urine PK analysis was limited due to lack of urine volume data. The urine concentrations were analyzed by the same model of plasma PK. The urine PK parameters were maximum urine concentration (urine Cmax), time to maximum urine concentration (urine Tmax), area under the drug concentration–time curve of urine (urine AUC) and the terminal half-life (urine t_1/2_) and elimination rate constant (Kel) of urine.

### 2.4. Statistical Analysis

The PK parameters were presented as mean ± standard error (SE). The parameters of 3 different Fosfomycin dosages were tested for normal distribution using the Shapiro–Wilk test. Tmax and F values were analyzed using Wilcoxon’s rank-sum test due to its non-normal distribution data. The AUC, Cmax, t_1/2β_ and Kel were assessed using the post hoc Bonferroni test. Differences were considered significant when *p*-value was < 0.05. The statistical software, SPSS 22.0 program (IBM Co., Chicago, Illinois, IL, USA) was used to analyze PK data. Graphs of Fosfomycin concentrations vs. time were plotted using Graphpad Prism 8.0 (GraphPad Software, San Diego, California, CA, USA).

## 3. Results

### 3.1. Animal Procedure

Six male beagle dogs were assigned to 3 different treatments: single oral Fosfomycin tromethamine at 40 mg/kg (treatment 1) and 80 mg/kg (treatment 2), and single intravenous injection of Fosfomycin disodium at 57 mg/kg (treatment 3), with 7 days washout period between each treatment. For treatment 1 and 2, plasma samples of each treatment were successfully collected from all dogs, while urine samples were collected from only five dogs for each treatment due to errors during the sample collection processes. For treatment 3, plasma and urine samples from one dog were excluded from the study because of a sample collection problem.

Loose stool was observed in two dogs (*n* = 2/6) after receiving oral Fosfomycin tromethamine at 40 mg/kg, three dogs (*n* = 3/6) after receiving 80 mg/kg, and one dog (*n* = 1/6) after receiving Fosfomycin disodium 57 mg/kg intravenously. All dogs had normal appetites and no other adverse effects were found during the study.

### 3.2. Method Validation

**Figure 1:**
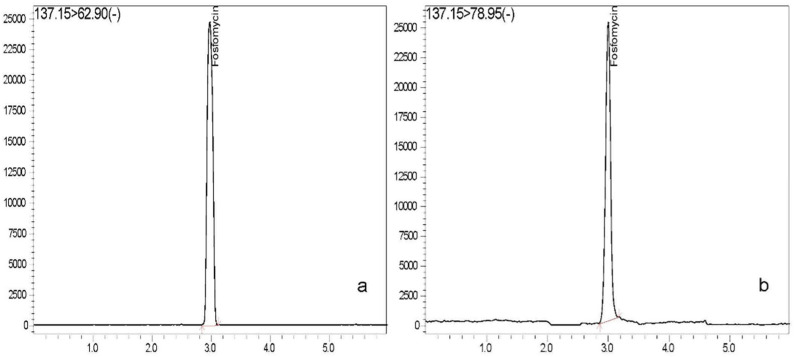
Chromatograms of Fosfomycin in plasma sample and urine sample. The MRM transitions were *m*/*z* 137.15 → 62.90 in plasma sample (**a**) and 137.15 → 78.95 in urine sample (**b**). (-) = negative ion mode.

For selectivity, no significant interference was detected at the retention time for Fosfomycin and IS in all of the plasma and urine batches. The chromatograms for Fosfomycin in plasma and urine are presented in Figure 1. Retention time of Fosfomycin and tadalafil (IS) was approximately 3.0 and 5.3 min in plasma and 3.0 and 5.0 min in urine, respectively. The total run time of both analyses was 6 min.

From the three batch run, the average correlation coefficient (R^2^) was 0.99477 ± 0.0002 in plasma and 0.99585 ± 0.0023 in urine. The percentage deviations of back calculated concentrations from nominal values ranged from −9.446 to 5.406 and −6.947 to 5.107 in plasma and urine, respectively. The results of calibration curves were within the acceptable range, demonstrating good linearity, accuracy, and precision.

The intra and inter batch of accuracy and precision are illustrated in Table 1. The inter batch of accuracy and precision was conducted in three different days. The percentages of accuracy of all QC samples (LLOQ, LQC, MQC and HQC) were within the acceptable criteria (85–115%), while the precision values of the LQC, MQC and HQC samples were <15%, except for the LLQC sample, which was <20%. Thus, the method was accurate and precise.

The mean recovery for the LQC and HQC samples was 70.32% and 77.17% for plasma, respectively and 93.89% and 90.35% for urine, respectively. The results were consistent, reproducible, and precise at ≤6.465%CV for plasma and ≤7.664%CV for urine.

The results of dilution integrity, shown in Table 2, demonstrated that study samples with concentrations above the ULOQ can be diluted, analyzed, and produce an accurate value. The accuracy and precision of all samples were within the acceptance criteria of 85–115% and ≤15%, respectively. Thus, the dilution did not affect the accuracy of the concentrations of the samples.

In the carry-over test, the extracted blank samples were injected after the extracted ULOQ, where no significant peak was detected at the retention time for Fosfomycin and IS, suggesting that no carry-over was present in the system.

For stability, the freeze and thaw of LQC and HQC samples demonstrated the stability of Fosfomycin up to three freeze–thaw cycles for plasma samples and up to five freeze–thaw cycles for urine samples. The percent deviation of LQC and HQC samples were 1.950% and −0.504% for plasma samples and 1.478% and 2.787% for urine samples, respectively, while the results of post-preparative stability demonstrated that the extracted samples were stable for 24 h at 15 °C in the auto-sampler. The percent deviation of post-preparative at LQC and HQC samples were −1.928% and −0.093% for plasma samples and −3.415% and 1.200% for urine samples, respectively. All the results presented no degradation of Fosfomycin during the analytical process.

### 3.3. Calibration Curve

The concentrations of Fosfomycin in plasma and urine samples were determined using the calibration curve of 0.050–140 µg/mL for plasma and 1.000–1000 µg/mL for urine, respectively. In this study, plasma Fosfomycin concentrations ranged from 0.053 µg/mL to 468.426 µg/mL, while urine Fosfomycin concentrations were from 1.188 µg/mL to 34,127.142 µg/mL.

When the analyzed sample concentration was above the upper limit of quantification (ULOQ: 140.00 µg/mL for plasma and 1000.00 µg/mL for urine), the sample was diluted with a blank of either plasma or urine at the appropriate ratios (1:5 for plasma and 1:10, 1:20 or 1:50 for urine) and back calculated by using the dilution factor as in the dilution integrity method of validation.

### 3.4. Pharmacokinetic Analysis

#### 3.4.1. Fosfomycin Concentrations in Plasma

**Figure 2:**
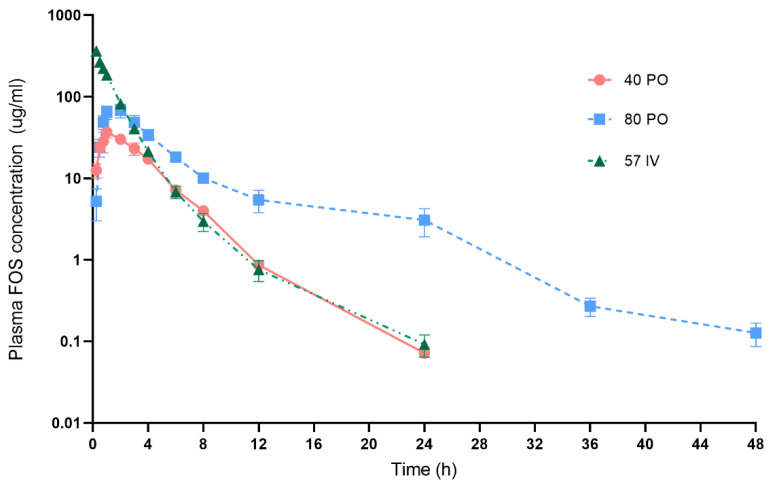
Mean (±SE) plasma concentrations of Fosfomycin in dogs. Drug concentrations after oral administrations of Fosfomycin tromethamine at 40 mg/kg (circle symbols) and 80 mg/kg (square symbols) (*n* = 6/treatment) and intravenous administration (IV) of Fosfomycin disodium at 57 mg/kg (triangle symbols) (*n* = 5). The y axis is in the log scale.

Fosfomycin plasma concentration–time curves after three different dosing regimens are presented in Figure 2. After receiving oral Fosfomycin at 40 mg/kg, plasma Fosfomycin concentrations were >10 µg/mL at the first 15 min and reached their peak within 1–2 h in all six dogs. Then, drug concentrations were dropped to <1 µg/mL after 12 h post-dose. Fosfomycin levels in plasma were quantified from 15 min up to 24 h. After 24 h, the drug levels were below the limit of quantitation (BLQ). At 80 mg/kg PO, the highest drug concentrations in plasma were detected at 1 h in 2 dogs and at 2 h in 4 dogs. Mean ± SE of Fosfomycin levels at 12 h were 5.45 ± 3.75 µg/mL. Half of all subjects (*n* = 3/6) had measurable Fosfomycin concentrations at 48 h. Fosfomycin concentrations were quantified from 15 min up to 48 h m/z. While at 57 mg/kg IV administration, almost of subjects (*n* = 4/5) had plasma Fosfomycin concentrations <1 µg/mL after 12 h and Fosfomycin concentrations of all 5 dogs were BLQ after 24 h *m/z*.

#### 3.4.2. Pharmacokinetic Parameters of Fosfomycin in Plasma

The pharmacokinetic parameters of Fosfomycin in dog plasma are presented in Table 3. The percent of oral bioavailability (F) of Fosfomycin tromethamine at 40 and 80 mg/kg relative to 57 mg/kg IV Fosfomycin disodium were not significantly different from each other (*p* = 0.146). The terminal half-life (t_1/2β_) of oral Fosfomycin tromethamine were significantly longer than Fosfomycin disodium IV (*p* < 0.05). The area under the concentration–time curve (AUC) values increased approximately 2.5 folds with a 2 fold increase in oral dose (40 to 80 mg/kg). The mean of Cmax from IV administration (approximately 364 µg/mL) was significantly higher than that from oral administration (approximately 34 and 66 µg/mL) (*p* < 0.05).

#### 3.4.3. Fosfomycin Concentrations in Urine

**Figure 3:**
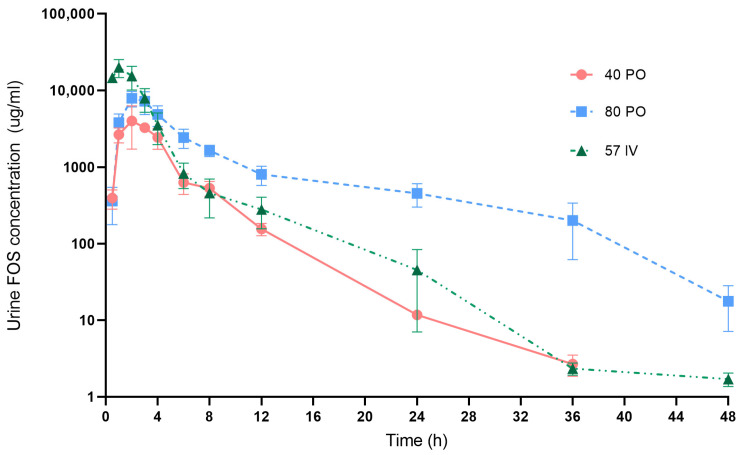
Mean (±SE) urine concentrations of Fosfomycin in dogs. Drug concentrations after oral administrations of Fosfomycin tromethamine at 40 mg/kg (circle symbols), 80 mg/kg (square symbols) and intravenous administration of Fosfomycin disodium at 57 mg/kg (triangle symbols) in dogs (*n* = 5/dosing group). The y axis is in the log scale.

Fosfomycin urine concentration-time curve after 3 different dosing regimens are presented in Figure 3. Fosfomycin concentrations in urine of all treatments were much (more than 100×) higher than those in plasma. After oral administration at 40 mg/kg, Fosfomycin concentrations were detected in urine within 30 min and increased to maximum concentration within 1–3 h post-dose in all 5 dogs. Mean ± SE of Fosfomycin levels at 4, 8 and 12 h were 2443.02 ± 737.26, 530.81 ± 117.82 and 155.60 ± 28.28 µg/mL, respectively. Almost of subjects (*n* = 4/5) had urine Fosfomycin concentrations >100 µg/mL at 12 h. From this dosage, Fosfomycin levels were quantified from 30 min up to 36 h after drug administration.

After oral Fosfomycin administration at 80 mg/kg, the highest concentrations were detected within 2–4 h. Mean ± SE of Fosfomycin levels at 4, 8 and 12 h were 4837.02 ± 1443.11, 1647.75 ± 272.28 and 801.59 ± 224.12 µg/mL, respectively. Fosfomycin concentrations remained above 100 µg/mL at 24 h in all five dogs. At 36 h, two dogs (*n* = 2/5) still had Fosfomycin levels >100 µg/mL. Urine Fosfomycin levels were quantified from 30 min up to 48 h post-dose.

In dogs receiving Fosfomycin at 57 mg/kg IV, the peak level was present at 1–2 h. Mean ± SE at 4, 8 and 12 h were 3520.34 ± 1558.54, 457.57 ± 241.11 and 280.42 ± 124.03 µg/mL, respectively. Four dogs (*n* = 4/5) had urine Fosfomycin concentrations >100 µg/mL at 12 h. Fosfomycin levels were quantified from 30 min up to 48 h after IV administration

#### 3.4.4. Pharmacokinetic Parameters of Fosfomycin in Urine

The pharmacokinetic parameters of Fosfomycin tromethamine in canine urine are exhibited in Table 4. The mean of urine Cmax following oral administrations at 40 and 80 mg/kg was not significantly different (*p* = 0.225). Urine t_1/2_ after receiving Fosfomycin orally was not significantly longer than for Fosfomycin IV injection (*p* = 0.311). The area under the concentration–time curve (AUC) values increased approximately 3 fold with a 2 fold increase in oral dose (40 to 80 mg/kg PO).

## 4. Discussion

Tromethamine salt has been widely recognized as effective in improving the oral absorption of Fosfomycin, while the other oral formulation, Fosfomycin calcium salt, exhibits low bioavailability in humans (12%) [23] and animals, such as pigs (20%) [37]. Fosfomycin tromethamine is currently favored over Fosfomycin calcium due to its greater bioavailability (F) [16,22]. However, the available information on the use of Fosfomycin tromethamine in dogs is limited. Empirical dosages have been noted at 75–150 mg/kg (equivalent to Fosfomycin 40–80 mg/kg) every 12 h [33] or 40 mg/kg every 12 h [27]. In dogs, Fosfomycin has been reported as low toxicity and well-tolerated up to 120–200 mg/kg [33]. The common adverse effect is GI upset, including diarrhea, and less appetite [27] while, in humans, the recommended dosage is 3 g as a single dose for UTI treatment due to its high concentrations (>128 µg/mL) in urine for 24–48 h [38]. To test the theory of using oral Fosfomycin tromethamine in dogs, especially for UTI treatment, the authors decided to study Fosfomycin tromethamine concentrations in canine plasma and urine after oral administration at 40 and 80 mg/kg. The data from two different dosages will aid in determining the appropriate dosage for clinical treatments. In this study, Fosfomycin disodium was used as the reference IV for oral bioavailability analysis and disodium salt is the only injectable Fosfomycin commercially available, while tromethamine is the oral form for Fosfomycin.

In this study, two dogs with single oral Fosfomycin tromethamine at 40 mg/kg had loose stool 1–2 times within 10 h after drug administration, whereas three dogs with 80 mg/kg PO also had loose stool for 2–3 times within 48 h post-administration. This adverse effect, loose stool, was similar to the previous study that 4 out of 12 dogs had diarrhea after receiving single oral Fosfomycin tromethamine at 80 mg/kg [11,28]. In humans, mild and transient diarrhea for 1 to 2 days have been reported as the most common side effect of oral Fosfomycin tromethamine [25]. The kidney and liver functions, as well as the urinalysis results, of all subjects in the three treatments, were normal, indicating no signs of renal toxicity. These findings are consistent with previous studies conducted on dogs, where a single dose of Fosfomycin was administered [34], as well as studies where Fosfomycin disodium was administered for three consecutive days [20]. However, this study was examined in dogs with a single dose that might not be sufficient to completely represent ‘s adverse effects. Investigating the potential side effects of drugs administered at multiple doses, particularly at high levels, including comprehensive toxicity analysis, should be conducted to validate the safety of Fosfomycin.

Oral Fosfomycin is partially absorbed in the small intestine through two mechanisms: a saturable carrier-mediated phosphate transport system and a non-saturable process with first-order kinetics [15]. When ingested with food, Fosfomycin is degraded by gastric acid. The level of gastric acidity and the rate of gastric emptying can impact the degree of Fosfomycin degradation and bioavailability [14,15,16]. Based on the previous research, Fosfomycin tromethamine should be administered on an empty stomach as food may decrease its absorption rate [24,39]. Therefore, all dogs in this study received oral Fosfomycin tromethamine for at least 30 min before a meal. In our study, Fosfomycin was detected in plasma at 15 min after oral administration, indicating its rapid oral absorption. The bioavailability (F) of oral Fosfomycin tromethamine was approximately 38–45%. While the previous study reported that F of oral Fosfomycin disodium (40 mg/kg) was 29% [20]. The higher F in this study may relate to tromethamine salt. Tromethamine salt has a role as a buffer to elevate the pH levels that could slow acid-catalyzed hydrolysis in the stomach [16]. Our finding confirm the suitability of Fosfomycin with tromethamine salt for oral administration in dogs. However, this conclusion was drawn from the analysis of two different Fosfomycin formulations. Further research on Fosfomycin calcium in dogs would broaden the data for comparing oral Fosfomycin formulations. Furthermore, the amount of absorbed drug depends on GI activities and interrupting factors. All dogs in this study were healthy, fasted and did not receive other drugs during the experiment. Testing with different conditions that affect absorption may provide further data.

Plasma Cmax and AUC from dogs receiving oral Fosfomycin tromethamine increased approximately 2 and 2.5 fold when a dose of Fosfomycin was increased 2 fold (40 to 80 mg/kg PO). This observation may imply the presence of linearity and proportionality properties. These findings were consistent with the earlier study in dogs that the plasma Cmax increased from 5.20 ± 0.4 µg/mL to 10.84 ± 0.5 µg/mL and AUC_0–24_ increased from 22.5 ± 2.24 µg/mL to 48.72 ± 2.87 µg/mL when doses of Fosfomycin disodium were doubled (40 to 80 mg/kg PO) [20]. Additionally, as the dose of oral Fosfomycin tromethamine increased (40 to 80 mg/kg PO), the plasma Tmax was extended from approximately 1.0 h to 2.7 h. At 80 mg/kg, variations in both Cmax and Tmax increased. These findings may be attributed to individual differences in absorption due to the fact that Fosfomycin is absorbed through a saturable carrier-mediated phosphate transport system in a first-order absorption process in the gastrointestinal tract [15]. The high dose may require a longer time for absorption. Although Fosfomycin at 80 mg/kg PO can extend drug levels, the risks of adverse effects such as loose stool may be increased dependent of dose. Based on our study, the numbers of dogs with loose stool after receiving Fosfomycin tromethamine increased (from two to three dogs) when doubled doses (40 to 80 mg/kg PO) were given. However, this study examined the effects of a single and two different doses of Fosfomycin. A multiple doses study should be investigated to expand information om dosages.

The volume of distribution of the central compartment (Vd) was close to the parameters reported in the early study of Fosfomycin disodium at a dose of 40 mg/kg IV (560 ± 27 mL/kg) in dogs [20]. This finding indicates that Fosfomycin may have low-moderate distribution to tissues in dogs [40]. Most of the Fosfomycin was excreted by glomerular filtration, while non-renal clearance was very rare. The total clearance of Fosfomycin aligned with the glomerular filtration rate [29]. In dogs, the glomerular filtration rate ranged from 2–5 mL/min/kg [41]. The clearance (Cl) values in this study were within ranges of the parameters reported in the previous study. Boothe and Hubka (2011) reported that the Cl of Fosfomycin disodium at dose 40 mg/kg IV was at 210 ± 104 mL/kg × h [28]. However, an earlier study reported a Cl of 14.2 ± 1.37 mL/kg × h for Fosfomycin disodium at the same dose [20]. The disparity in Cl could be attributed to differences in individual glomerular filtration rate, variations in water intake volume, and urine output.

The plasma terminal half-life (t_1/2β_) of oral Fosfomycin tromethamine (approximately 2.7 h) was significantly longer than the t_1/2β_ of Fosfomycin disodium IV (approximately 1.9 h) (*p* < 0.05). This result corresponds closely to the previous study in dogs that t_1/2β_ of oral Fosfomycin disodium (approximately 2.2 h) was higher than that after IV administration (approximately 1.3 h) [20]. Moreover, in humans, a longer t_1/2_ of oral Fosfomycin tromethamine has also been reported [42]. The slow absorption may be attributed to the presence of a saturable carrier-mediated phosphate transport system in the absorption step [42].

Fosfomycin is mostly excreted unchanged in urine with approximately 40–50% of the dose found there [17,18,23,24]. In humans, Fosfomycin concentration in urine is usually high compared with its concentration in plasma [29,43,44]. From this study, urine Cmax after receiving oral Fosfomycin tromethamine at 40 and 80 mg/kg (4463.07 and 8784.93 µg/mL) was approximately 130 fold higher than the plasma Cmax (34.46 and 66.40 µg/mL) at the same doses. These results are consistent with the previous study in humans given a single oral dose of 3 g Fosfomycin tromethamine that the maximum urinary concentrations (1050.3–4378.9 µg/mL) were much higher than the plasma Cmax (17.5–47.7 µg/mL) [45]. Previously, Harada et al. (2020) examined the urine pharmacokinetics of Fosfomycin in dogs after administering oral Fosfomycin at 80 mg/kg. The means (± SE) of urine concentrations were 1348.2 ± 163.5, 1191.6 ± 260.2, and 661.1 ± 190.4 µg/mL at 0–4, 4–8, and 8–12 h, accordingly. These parameters were close to our results (the mean (± SE) of Fosfomycin concentrations at 8 and 12 h (1647.75 ± 272.28 and 801.59 ± 224.12 µg/mL, respectively) with 80 mg/kg Fosfomycin PO. These findings indicate that oral Fosfomycin tromethamine can achieve high concentrations in urine, high enough to have adequate efficacy for bacterial cystitis treatment in dogs as in humans.

The significant difference between oral administration and IV administration was not found in urine t_1/2_, unlike plasma t_1/2_, which may be a consequence of the variation of drug levels in urine. In this study, all dogs received water ad libitum. The difference in water intake may vary the urine output and urine PK parameters. Due to no data for urine volume, the urine PK analysis was limited.

The PK results indicate that oral Fosfomycin tromethamine has a low to moderate bioavailability. When the dose was doubled, the plasma and urine AUC and Cmax of oral Fosfomycin tromethamine increased. The peak urine concentration was over 100 times higher than its peak plasma concentration. These findings support the use of oral Fosfomycin tromethamine in the treatment of canine bacterial cystitis. However, the plasma concentrations may not be high enough to effectively inhibit bacteria with high minimum inhibitory concentration (MIC).

Pharmacokinetics or PK (drug concentrations and time profile) and pharmacodynamics or PD (antibacterial activities, i.e., minimum inhibitory concentration or MIC) have been integrated to evaluate the efficacy of an antibacterial drug via PK/PD ratios [46]. According to canine urine PK in this study, urine Cmax was approximately 4463 and 8784 µg/mL after administration of oral Fosfomycin at 40 and 80 mg/kg, respectively. Earlier studies have published the in vitro pharmacodynamics of Fosfomycin against bacterial pathogens of dogs. Hubka and Boothe (2011) reported that MIC of Fosfomycin against *E. coli* from canine UTI ranged from 0.25 to 196 µg/mL, with MIC50 and MIC90 of 1 and 3 µg/mL, respectively [10]. Based on these in vitro PD data, urine Cmax/MIC50 and urine Cmax/MIC90 ratios would be greater than 1000. Thus, oral administration of Fosfomycin provides high urine Fosfomycin concentrations, enough to kill uro-pathogenic bacteria.

However, the in vitro antimicrobial activity may not clarify the antibacterial exposure and drug efficacy [19,42]. Many conditions in bacterial culture were different from that of an animal body. The in vivo study of a neutropenic murine thigh infection model using a Fosfomycin disodium injection demonstrated that the AUC/MIC ratio had the greatest link with Fosfomycin efficacy. The net stasis and 1-log kill for Enterobacteriaceae bacteria were 23 and 83, respectively [47]. If these results are applied to our study, the plasma AUC0–24 of 150 mg/kg PO (approximately 343 µg × h/mL) could achieve net stasis and 1-log kill for Enterobacteriaceae isolates with MIC ≤ 8 µg/mL and ≤4 µg/mL, respectively. However, the urine AUC0–24 of 150 mg/kg PO (approximately 42,779.13 µg × h/mL) could achieve net stasis and a 1-log kill with MIC ≤ 256 µg/mL. Based on Clinical and Laboratory Standards Institute (CLSI) guidelines, the susceptibility MIC breakpoint for oral Fosfomycin tromethamine (human) is ≤64 µg/mL [48]. From a target PK/PD index (AUC/MIC) of 23, oral administration of Fosfomycin tromethamine could provide high urine Fosfomycin concentrations, enough to inhibit susceptible bacterial with MIC ≤ 64 µg/mL. On the other hand, the plasma AUC values may not be high enough to inhibit bacterial isolates with MIC ≥ 8 µg/mL. Therefore, oral Fosfomycin tromethamine may be a good choice for bacterial cystitis but not for other systemic bacterial infections in dogs.

Furthermore, PD study of Fosfomycin suggests that the pH level affects its antibacterial activity against *E. coli* isolates [49]. Decreasing pH from 7.0 to 6.0 could improve its antibacterial activity. The acidification of the growth medium could be a crucial factor in the efficacy of Fosfomycin [49]. The pH should be adjusted appropriately to mimic the conditions of a natural bacterial infection. Clinical experiments for Fosfomycin efficacy in dogs with UTI may complete the PK/PD data. Antibacterial susceptibility testing should be performed for rational drug selection. Fosfomycin may be prescribed to dogs with bacterial cystitis when the causative bacterial pathogens are susceptible and other treatment options have proven ineffective. However, the use of Fosfomycin in dogs is considered extra-labelled and should be closely monitored by veterinary pharmacology experts. This cautious approach is necessary due to concerns related to public health and antimicrobial resistance.

The results from our study provide plasma and urine pharmacokinetic parameters, including concentration-time profiles of Fosfomycin in dogs, after oral administration of Fosfomycin tromethamine and IV injection of Fosfomycin disodium. Although Fosfomycin is rarely used in dogs, its pharmacokinetics support the clinical use of Fosfomycin in canine UTI, especially in cases of multidrug resistance when other antibacterial drugs are not suitable.

## 5. Conclusions

Fosfomycin tromethamine was absorbed after oral administration with bioavailability of approximately 38–45%. Peak urine concentration of Fosfomycin was more than 100 times greater than its peak plasma concentration, supporting its use in the treatment of canine urinary tract infection. However, this study was limited to single dose administration in healthy dogs, thus further study with multiple dose administration should be performed to extend PK information. Additionally, PK/PD research with multidrug resistant bacteria from canine UTI may be useful to confirm the efficacy of Fosfomycin tromethamine in dogs.

## Figures and Tables

**Table 1:** The intra and inter batch accuracy and precision of Fosfomycin in plasma and urine at LLOQ, low, medium, and high concentrations.

	Concentration of Fosfomycin in Plasma and Urine (µg/mL)							
Fosfomycin	LLOQ		LQC		MQC		HQC	
	Plasma	Urine	Plasma	Urine	Plasma	Urine	Plasma	Urine
Nominal value (µg/mL)	0.05	1	0.75	3	55	350	110	750
Intra-batch								
Mean	0.048	1.14	0.73	2.93	52.30	353.28	98.76	691.36
SD	0.005	0.06	0.03	0.29	0.47	6.51	1.26	8.05
Precision (%CV)	10.29	5.39	4.10	9.72	0.91	1.84	1.27	1.17
%Accuracy	96.80	113.66	98.76	99.45	95.09	100.65	89.78	92.18
Inter-batch								
Mean	0.05	1.06	0.74	2.99	52.98	356.26	102.86	744.50
SD	0.003	0.07	0.005	0.07	0.89	2.82	5.07	48.95
Precision (%CV)	6.18	6.66	0.68	2.47	1.68	0.79	4.93	6.58
%Accuracy	99.07	105.84	99.52	101.25	96.32	101.50	93.51	99.27

LLOQ, lower limit of quantification; LQC, low quality control; MQC, medium quality control; HQC, high quality control. Accuracy should be within 85–115% of the nominal value for all QC samples, except for LLOQ within 80–120%. Precision (%CV) should be ≤15% for all QC samples, except that LLOQ should be ≤20%.

**Table 2:** Dilution integrity test of Fosfomycin in plasma and urine.

	Plasma Samples		Urine Samples		Urine Samples		Urine Samples	
	1:5 Dilution		1:10 Dilution		1:20 Dilution		1:50 Dilution	
Fosfomycin	Back cal conc.	Back cal conc. × Factor	Back cal conc.	Back cal conc. × Factor	Back cal conc.	Back cal conc. × Factor	Back cal conc.	Back cal conc. × Factor
		(Factor = 6)		(Factor = 11)		(Factor = 21)		(Factor = 51)
Nominal value (µg/mL)	36.67	220.00	136.36	1499.99	71.43	1499.99	29.41	1499.99
Mean	34.22	205.31	145.96	1605.53	80.61	1692.73	28.74	1465.80
SD	1.24	7.43	4.37	48.11	8.20	172.14	1.37	69.79
Precision (%CV)	3.62	3.62	3.00	3.00	10.17	10.17	4.76	4.76
% Accuracy	93.32	93.32	107.04	107.04	112.85	112.85	97.72	97.72

Back cal conc., back calculation concentration. Accuracy should be within 85–115%. Precision (%CV) should be ≤15%.

**Table 3:** Canine plasma Fosfomycin pharmacokinetic parameters.

PK Parameters	Unit	IV	PO	
		57 mg/kg (*n* = 5)	40 mg/kg (*n* = 6)	80 mg/kg (*n* = 6)
		Mean ± SE	Mean ± SE	Mean ± SE
AUC_0–∞_	µg × h/mL	558.50 ± 42.33 ^bc^	145.75 ± 11.49 ^ac^	358.16 ± 24.62 ^ab^
AUC_0–t_	µg × h/mL	558.00 ± 42.31 ^bc^	145.47 ± 11.51 ^ac^	356.77 ± 24.49 ^ab^
AUC_0–24_	µg × h/mL	558.00 ± 42.31 ^bc^	145.47 ± 11.51 ^ac^	343.16 ± 24.27 ^ab^
F	%	-	38.55 ± 3.34	45.66 ± 2.64
Cmax	µg/mL	364.34 ± 36.88 ^bc^	34.46 ± 5.11 ^a^	66.40 ± 12.64 ^a^
Tmax	h	-	1.08 ± 0.20	2.67 ± 1.09
Vd	mL/kg	739.16± 73.39	-	-
Vd/F	mL/kg	-	2076.55 ± 205.39	1728.36± 130.17
Cl	mL/kg × h	181.86 ± 17.55	-	-
Cl/F	mL/kg × h	-	530.50 ± 40.65	475.35 ± 22.81
t_1/2ab_	h	-	0.43 ± 0.16	0.85 ± 0.35
t_1/2β_	h	1.91 ± 0.07 ^bc^	2.70 ± 0.12 ^a^	2.79 ± 0.09 ^a^
Ka	1/h	-	1.91 ± 0.44	1.31 ± 0.30
Kel	1/h	0.36 ± 0.01 ^bc^	0.26 ± 0.01 ^a^	0.25 ± 0.01 ^a^

AUC_0–∞_ = the area under the concentration–time curve from time zero to time infinity; AUC_0–t_ = the area under the concentration–time curve from time zero to the time of last detectable concentration; AUC_0–24_ = the area under the concentration–time curve from time zero to the time 24 h; µg × h/mL = μg × h/mL; F = bioavailability; Cmax = maximum concentration; Tmax = time of maximum concentration; Vd = apparent volume of distribution of the central compartment; Vd/F = apparent volume of distribution associated with F (bioavailability); Cl = clearance; Cl/F = apparent clearance associated with F (bioavailability); mL/kg × h = mL/kg × h; t_1/2ab_ = absorption half-life; t_1/2β_ = terminal half-life; Ka = absorption rate constant and Kel = elimination rate constant. ^a^ Significantly different from Fosfomycin 57 mg/kg IV (*p* < 0.05). ^b^ Significantly different from Fosfomycin 40 mg/kg PO (*p* < 0.05). ^c^ Significantly different from Fosfomycin 80 mg/kg PO (*p* < 0.05).

**Table 4:** Canine urine Fosfomycin pharmacokinetic parameters.

PK Parameters	Unit	IV	PO	
		57 mg/kg (*n* = 5) Mean ± SE	40 mg/kg (*n* = 5) Mean ± SE	80 mg/kg (*n* = 5) Mean ± SE
AUC_0–∞_	µg × h/mL	57,946.14 ± 14,353.09 ^b^	15,471.15 ± 2363.53 ^ac^	46,959.06 ± 4237.72 ^b^
AUC_0–t_	µg × h/mL	57,898.06 ± 14,373.60 ^b^	15,450.45 ± 2366.17 ^ac^	46,757.53 ± 4293.95 ^b^
AUC_0–24_	µg × h/mL	57,804.83 ± 14,300.92 ^b^	15,390.22 ± 2361.07 ^ac^	42,779.13 ± 5032.90 ^b^
Urine Cmax	µg/mL	23,489.60 ± 4472.98 ^bc^	4463.07 ± 987.84 ^a^	8784.93 ± 2303.46 ^a^
Urine Tmax	h	1.10 ± 0.24 ^c^	2.00 ± 0.32	2.60 ± 0.4 ^a^
Urine t_1/2_	h	2.04 ± 0.26	2.94 ± 0.52	3.01 ± 0.53
Urine Kel	1/h	0.36 ± 0.04	0.27 ± 0.05	0.26 ± 0.05

AUC_0–∞_ = the area under the concentration–time curve from time zero to time infinity; AUC_0–t_ = the area under the concentration–time curve from time zero to the time of last detectable concentration; AUC_0–24_ = the area under the concentration–time curve from time zero to the time 24 h; µg × h/mL = μg × h/mL; Urine Cmax = maximum concentration in urine; Urine Tmax = time of maximum concentration in urine; Urine t_1/2_ = terminal half-life of urine and Urine Kel = elimination rate constant of urine. ^a^ Significantly different from Fosfomycin 57 mg/kg IV (*p* < 0.05). ^b^ Significantly different from Fosfomycin 40 mg/kg PO (*p* < 0.05). ^c^ Significantly different from Fosfomycin 80 mg/kg PO (*p* < 0.05).

## Data Availability

The data presented in this study is available upon reasonable request.
